# The Effect of a Cellular-Enabled Glucose Meter on Glucose Control for Patients With Diabetes: Prospective Pre-Post Study

**DOI:** 10.2196/14799

**Published:** 2019-10-07

**Authors:** Jennifer B Bollyky, Stephanie T Melton, Tong Xu, Stefanie L Painter, Brian Knox

**Affiliations:** 1 Stanford University School of Medicine Stanford, CA United States; 2 University of South Florida Florida, CA United States; 3 Livongo Health Mountain View, CA United States

**Keywords:** diabetes, blood glucose, blood glucose meter

## Abstract

**Background:**

Diabetes is a global epidemic affecting approximately 30 million people in the United States. The World Health Organization recommends using technology and telecommunications to improve health care delivery and disease management. The Livongo for Diabetes Program offers a remote monitoring technology with Certified Diabetes Educator outreach.

**Objective:**

The purpose of this study was to examine health outcomes measured by changes in HbA_1c_, in time in target blood glucose range, and in depression symptoms for patients enrolled in a remote digital diabetes management program in a Diabetes Center of Excellence setting.

**Methods:**

The impact of the Livongo for Diabetes program on hemoglobin A_1c_ (HbA_1c_), blood glucose ranges, and depression screening survey results (Patient Health Questionnaire-2 [PHQ-2]) were assessed over 12 months in a prospective cohort recruited from the University of South Florida Health Diabetes Home for Healthy Living. Any patient ≥18 years old with a diagnosis of diabetes was approached for voluntary inclusion into the program. The analysis was a pre-post design for those members enrolled in the study. Data was collected at outpatient clinic visits and remotely through the Livongo glucose meter.

**Results:**

A total of 86 adults were enrolled into the Livongo for Diabetes program, with 49% (42/86) female, an average age of 50 (SD 15) years, 56% (48/86) with type 2 diabetes mellitus, and 69% (59/86) with insulin use. The mean HbA_1c_ drop amongst the group was 0.66% (*P*=.17), with all participants showing a decline in HbA_1c_ at 12 months. A 17% decrease of blood glucose checks <70 mg/dL occurred concurrently. Participants with type 2 diabetes not using insulin had blood glucose values within target range (70-180 mg/dL) 89% of the time. Participants with type 2 diabetes using insulin were in target range 68% of the time, and type 1 diabetes 58% of the time. Average PHQ-2 scores decreased by 0.56 points during the study period.

**Conclusions:**

Participants provided with a cellular-enabled blood glucose meter with real-time feedback and access to coaching from a certified diabetes educator in an outpatient clinical setting experienced improved mean glucose values and fewer episodes of hypoglycemia relative to the start of the program.

## Introduction

Diabetes mellitus is now considered an epidemic, as global prevalence approaches 500 million people with the disease [1]. Approximately 30 million people have diabetes in the United States, and 84 million are at a high risk of developing the disease within 5 years [2]. Poor control of diabetes is shown to be related to a lack of knowledge around blood glucose (BG) monitoring, proper nutrition, and medication self-management [3]. A lack of consistent access to educational resources and episodic communication with providers may be responsible for poor outcomes in daily self-management [4,5].

In an effort to improve diabetes care and outcomes, the World Health Organization (WHO) recommends the use of mobile telecommunications in the health care setting to improve health care delivery and disease management [6]. Multimedia technologies have also been shown to increase patient satisfaction, access, adherence, and cost effectiveness [7-10]. Specifically, when electronic glucose monitoring is combined with personalized feedback or expert coaching, Hemoglobin A_1_c (HbA_1c_) levels improve significantly [11-16]. Access to a cellular-connected glucose monitor with real-time feedback from certified diabetes educators (CDEs) decreased the likelihood of experiencing hypoglycemia or hyperglycemia up to 18% monthly, and it also decreased HbA_1c_ by 1% every 3 months and nearly 2% over 12 months [11,13,15]. Technology-facilitated care has also been significantly associated with depression remission, depression free days, and increased satisfaction of care [17,18].

The purpose of this study was to examine health outcomes measured by changes in HbA_1c_, time in the target BG range, and depression symptoms for patients enrolled in a remote digital diabetes management program in a Diabetes Center of Excellence setting.

## Methods

### Study Design

This was a prospective study that investigated the impact of the Livongo for Diabetes program on HbA_1c_ and the proportion of BG checks in range for patients with diabetes mellitus at the University of South Florida Diabetes Home for Healthy Living (USF DHHL). The Livongo for Diabetes program is a digital chronic condition management program that combines: (1) a Food and Drug Administration–cleared, cellular-enabled, two-way messaging glucometer that measures blood glucose and delivers personalized digital coaching messages (see Multimedia Appendix 1); (2) free unlimited blood glucose test strips; and (3) unlimited access to CDEs for goal setting and behavioral and lifestyle education based on the American Diabetes Association’s (ADA) Standards of Medical Care and the American Association of Diabetes Educator’s (AADE) Diabetes Education Prompt Deck and Educator Guide [19,20].

Personalized digital coaching methods are delivered algorithmically on the meter to members based on diabetes type, medication use, and clinical guidelines. Immediately following each BG check, members receive context-specific feedback based on the BG value measured, as well as BG trends and patterns established with repeated meter usage. This feedback is delivered through messages less than 140 characters in length and based on ADA and AADE recommendations.

The CDEs also provided 24 hours a day, 7 days a week, 365 days a year call support for members with BG readings of <50 mg/dL or >400 mg/dL, within 3 minutes of transmitted blood glucose, to provide ADA-recommended, nonmedication-related interventions to effect their BG (ie, “drink 8 ounces of orange juice to bring your BG values up and recheck BG in 15 minutes”).

In addition to the Livongo glucometer and access to CDEs, participants had access to a mobile phone application on iOS and Android, and a web portal available through traditional web browsers that tracked historical BG readings, provided reminders for BG checking, and allowed members to send Health Summary Reports of BG readings to care providers, family members, and friends (see Multimedia Appendix 2).

### Participants

**Figure 1:**
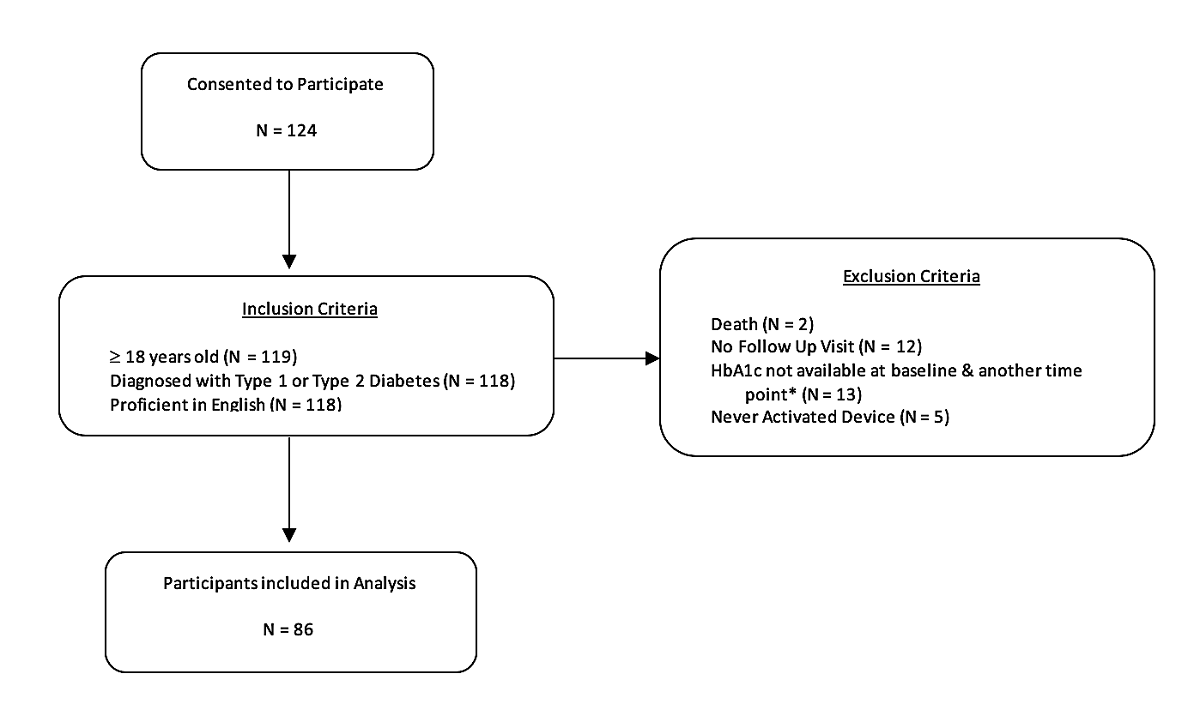
Study population. *Valid baseline hemoglobin A_1c_ (HbA_1c_) values defined as HbA_1c_ test taken within 90 days before registration date and 45 days after registration date.

A convenience sample of participants were enrolled from the USF DHHL clinic from February 2015 to February 2016. All participants recruited for the study were established patients of the clinic with elevated HbA_1c_ who were receiving their usual care. Patients were eligible if they were at least 18 years of age, diagnosed with type 1 or type 2 diabetes mellitus, and proficient in English. Patients were excluded from the study if they did not have a baseline HbA_1c_ value and at least one other HbA_1c_ value within the study period for comparison, did not have a follow-up visit, and never activated the device (Figure 1). In addition, patients who died during the study were also excluded due to unavailable health information because of closed medical records.

The study protocol was approved by the University of South Florida Institutional Review Board (Protocol #PRO00016476). Verbal and written informed consent were obtained prior to participant’s enrollment in the study. Study procedures were conducted in compliance with the Declaration of Helsinki.

### Measures

#### Blood Glucose

Blood glucose values were captured remotely in real-time from the cellular-connected Livongo glucose meter provided to participants. Target BG range was defined between 70 and 180 mg/dL. Additionally, BG values of <70 mg/dL and >180 mg/dL were defined as low and high, respectively.

#### Hemoglobin A_1c_

HbA_1c_ was measured at the DHHL clinic using the Siemens DCA Vantage Analyzer. Eligible study participants were required to have recorded HbA_1c_ values within 3 months prior to their Livongo program registration date and at least one subsequent HbA_1c_ value for comparison during the 12-month study period. HbA_1c_ was measured at every subsequent clinic visit as clinically indicated for one year.

#### Patient Health Questionnaire

Patient Health Questionnaire-2 (PHQ-2) is a validated, patient-reported outcome tool that assesses the frequency of depressed mood and anhedonia over the past two weeks as a screen for depression. The ADA recommends that providers consider annually screening all people living with diabetes for depression, as they have up to a 35% higher incidence of depressive symptoms than those without diabetes [15,21]. A PHQ-2 score ranges from 0-6, where a score of 3 or higher indicates further evaluation for depression should be pursued. Participants were asked PHQ-2 survey questions within one month of program enrollment and again at the end of the study period.

### Statistical Analysis

Summarizing statistics were computed for demographic characteristics. Outcome variables were computed between baseline and subsequent clinic visits. The nonparametric Wilcoxon rank sum test was used to compare continuous variables, and Fisher’s exact test was used for categorical data comparisons.

## Results

### Baseline Characteristics

Baseline characteristics are presented in Table 1. Nearly half of the participants were female, with a mean age of 50 (SD 15) years, and were diagnosed with type 2 diabetes (56%; 48/86).

**Table 1:** Demographic characteristics.

Characteristics		Type 1 diabetes (n=38)	Type 2 diabetes			Overall population (N=86)	
			Insulin use (n=21)	No insulin use (n=27)			
Gender, female, n (%)		20 (52.6)	12 (57.1)	10 (37)		42 (48.8)	
**Age (years)**							
	Mean (SD)	39.3 (11.3)	57.1 (12.1)	59 (12.1)		49.8 (15.0)	
	Median (IQR^a^)	38.0 (15.3)	55.0 (16.0)	62.0 (14.0)		49.5 (24.8)	
**Body mass index**							
	Mean (SD)	28.0 (5.3)	36.8 (10.7)	31.0 (4.4)		30.8 (7.2)	
	Median (IQR)	26.0 (7.4)	32.6 (6.7)	30.7 (4.1)		30.4 (6.6)	
**Race, n (%)**							
	White	6 (15.8)	1 (4.8)	2 (7.4)		9 (10.5)	
	Hispanic	1 (2.6)	0 (0)	0 (0)		1 (1.2)	
	Black	3 (7.9)	1 (4.8)	1 (3.7)		5 (5.8)	
	Other	28 (73.7)	19 (90.5)	24 (88.9)		71 (82.6)	
**Daily blood glucose checking frequency**							
	Mean (SD)	1.3 (1.3)	1.2 (1.0)	1.0 (0.9)		1.2 (1.1)	
	Median (IQR)	0.8 (1.8)	1.0 (1.2)	0.8 (1.0)		0.8 (1.6)	
**Insulin use, n (%)**							
	Once a day	29 (76.3)	19 (90.5)	0 (0)		48 (55.8)	
	More than once a day	9 (23.7)	2 (9.5)	0 (0)		11 (12.8)	
	No use	0 (0)	0 (0)	27 (100)		27 (31.4)	
**Self-reported blood pressure category, n (%)**							
	High	13 (34.2)	8 (38.1)	5 (18.5)		26 (30.2)	
	Normal	24 (63.2)	13 (61.9)	18 (66.7)		55 (64.0)	
	Unknown	1 (2.6)	0 (0)	4 (14.8)		5 (5.8)	
**Smoker, n (%)**							
	Never smoked	32 (84.2)	17 (81.0)	23 (85.2)		72 (83.7)	
	No, quit on given date	2 (5.3)	1 (4.8)	0 (0)		3 (3.5)	
	Yes, not trying to quit	4 (10.5)	3 (14.3)	4 (14.8)		11 (12.8)	

^a^IQR: interquartile range.

### Hemoglobin A_1c_

**Figure 2:**
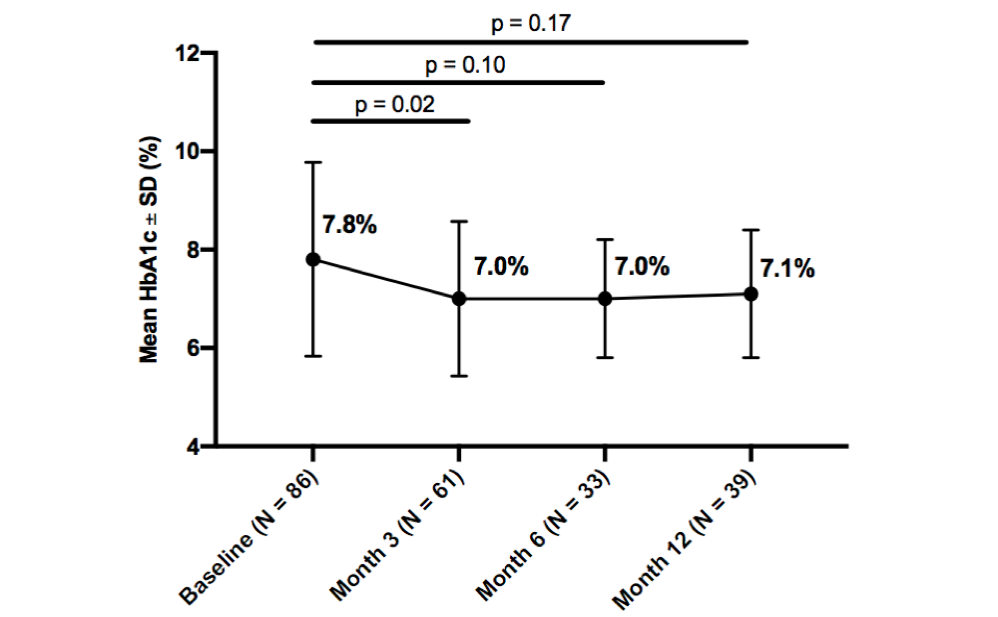
Change in hemoglobin A_1c_ (HbA_1c_) from baseline by timepoint for all participants.

**Figure 3:**
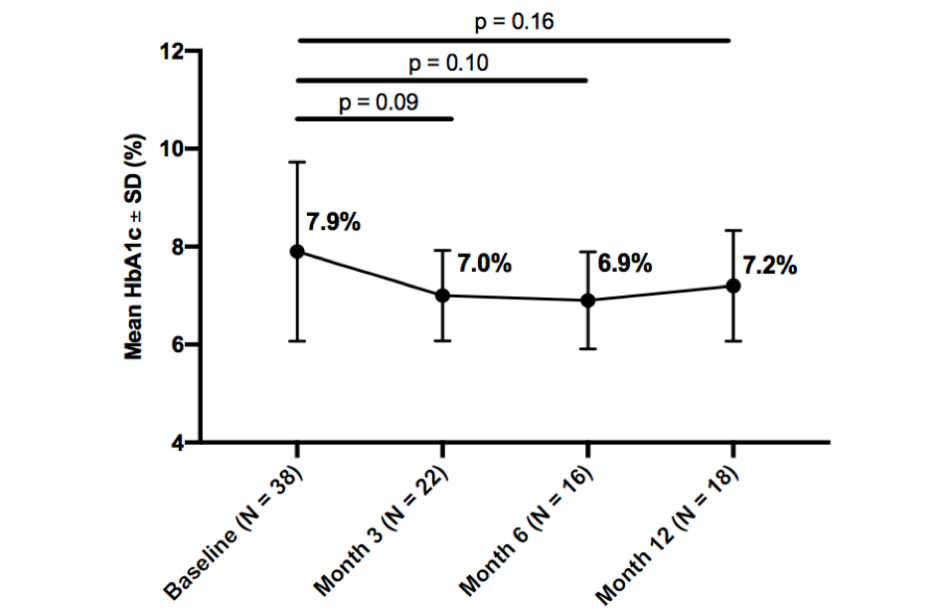
Change in hemoglobin A_1c_ (HbA_1c_) from baseline by timepoint for type 1 diabetes.

**Figure 4:**
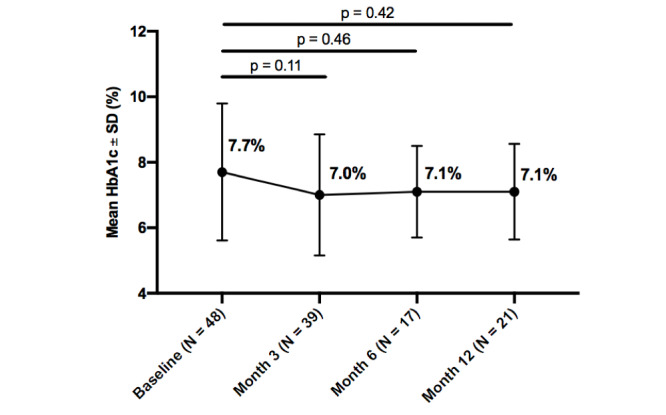
Change in hemoglobin A_1c_ (HbA_1c_) from baseline by timepoint for type 2 diabetes.

**Figure 5:**
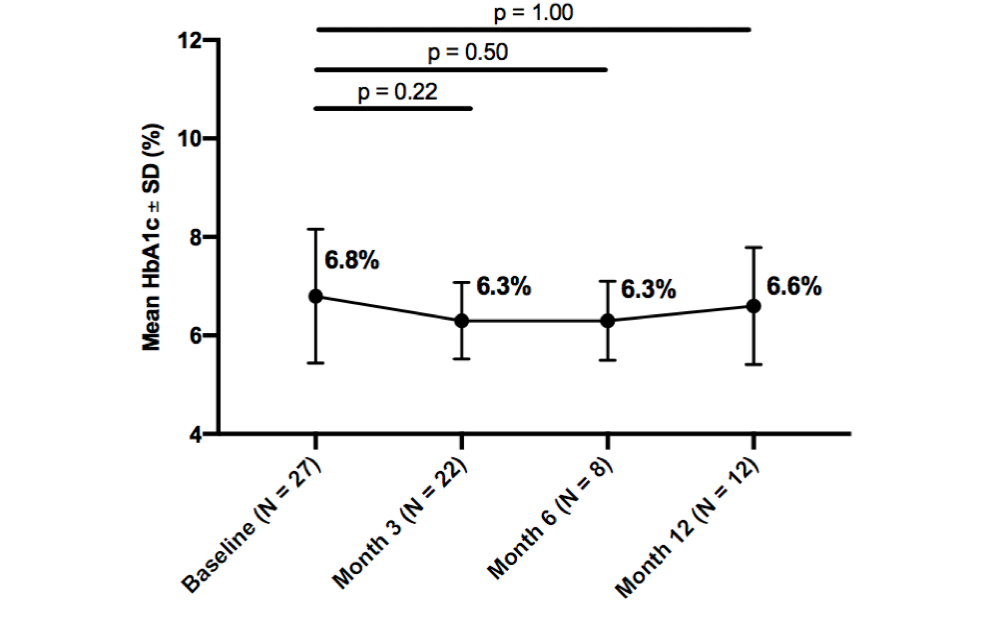
Change in hemoglobin A_1c_ (HbA_1c_) from baseline by timepoint for type 2 diabetes without insulin use.

**Figure 6:**
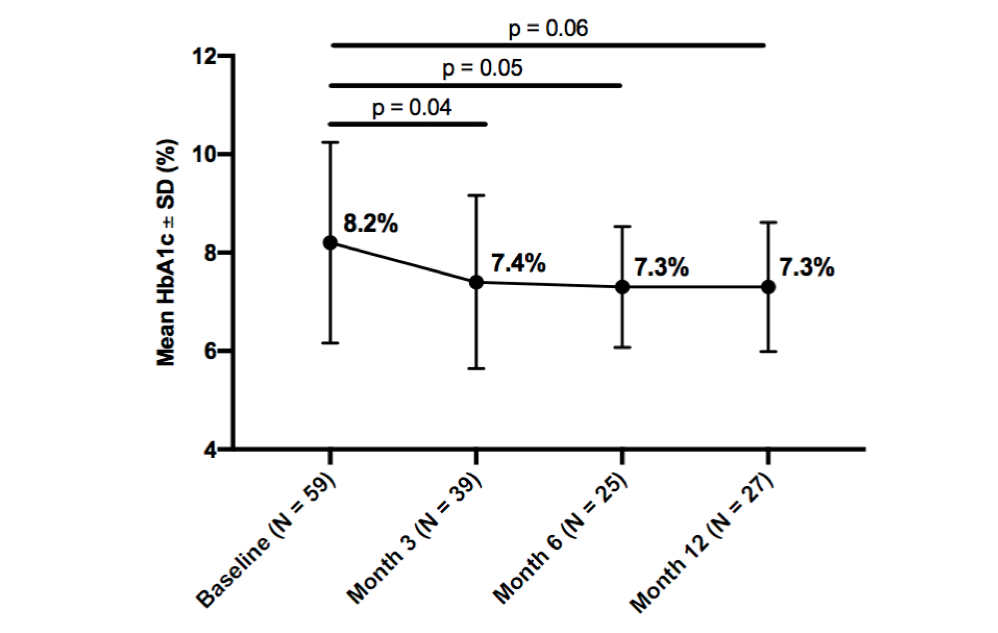
Change in hemoglobin A_1c_ (HBA_1c_) from baseline by timepoint for all participants with insulin use.

Mean HbA_1c_ improved from baseline in all participants throughout the intervention, and within each diabetes type. Statistically significant improvements were seen in all participants from baseline to 3 months (0.8%; *P*=.02). Additionally, insulin users, whether with type 1 or type 2 diabetes, experienced a greater decrease in HbA_1c_ than noninsulin users, at both 3 months (0.8%; *P*=.04) and 6 months (1.0%; *P*=.05). While HbA_1c_ improved from baseline to 12-months, it was not statistically significant at any time point for participants with type 1 diabetes, type 2 diabetes, or participants with no insulin usage, whether with type 1 or type 2 diabetes. Further details about changes in HbA_1c_ over the 12-month intervention by subgroups are reported in Figures 2-6.

### Percent of Blood Glucose Checks Within Target Range

Blood glucose values were analyzed by BG range categories, and by diabetes type, for all participants during the study period. Median BG checking frequency ranged from 0.8 to 1.0 times per day depending on diabetes type and insulin use. Participants with type 2 diabetes using insulin had the highest BG checking frequency at approximately 1.0 (SD 1.2) times per day, while participants with type 2 diabetes not using insulin checked an average of 0.8 (SD 1.0) times per day and participants with type 1 diabetes were at 0.8 (SD 1.8) checks per day.

Patients with type 2 diabetes not using insulin had the highest percentage of BG checks within the target range of 70-180mg/dL (89.0%; SD 15.9), compared to participants with type 2 diabetes on insulin (68.1%; SD 28.1) and type 1 diabetes (57.9%; SD 22.5) throughout the 12 months.

**Figure 7:**
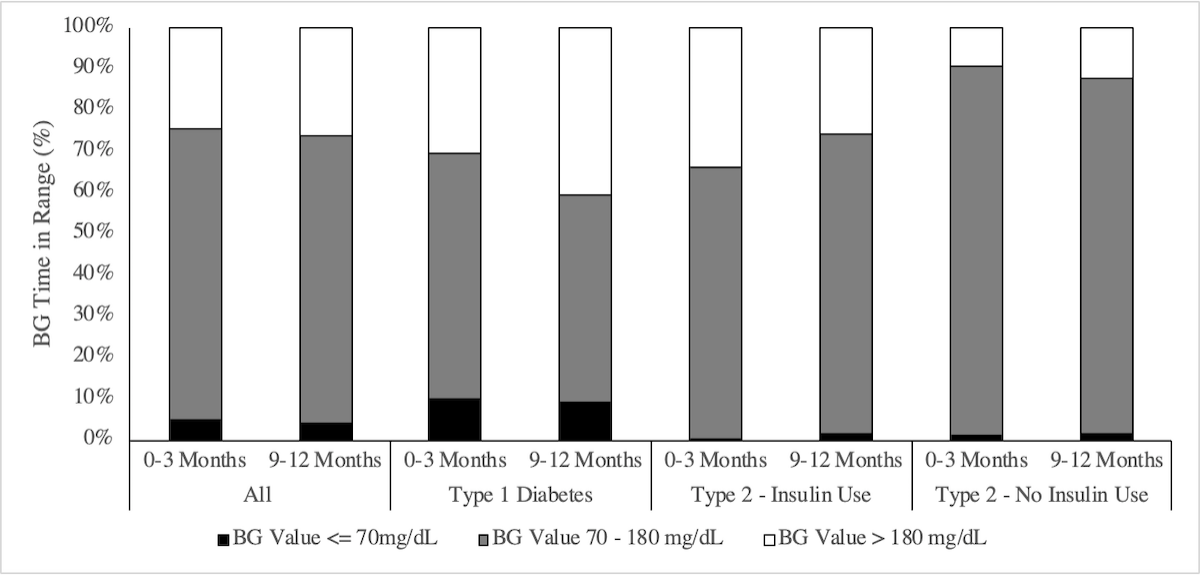
Percent blood glucose (BG) checks in range by diabetes type and insulin use.

When comparing percentage of BG checks in range in the first three months of the study versus the last three months, all participants decreased their percentage of BG checks that were <70 mg/dL from 4.9% to 4.1% (*P*=.56; see Figure 7). Though not statistically significant, participants with type 1 diabetes experienced a slight decrease in percentage of BG checks over 400 mg/dL from 1.8% to 1.5% (*P*=.81). Similarly, participants with type 2 diabetes using insulin had a nonsignificant decrease in percentage of BG checks greater than 180 mg/dL from 33.5% to 25.9% (*P*=.43). Participants with type 2 diabetes not using insulin showed no significant improvements for BG time in range. Further comparison of BG checks in range by diabetes type from 0-3 months to 9-12 months is shown in Multimedia Appendix 3.

### Depression Screening

Over the intervention period, there was a statistically significant decrease in mean PHQ-2 score (*P*=.04) among all participants. Average baseline PHQ-2 score (N=40) was 0.83. Postintervention score (N=19) was 0.26. While both baseline and postintervention scores were <3, showing an unlikelihood for depression symptoms, participants still showed a statistically significant decrease from baseline to 12 months. When analyzing PHQ-2 score by diabetes type and insulin usage, participants using insulin showed a statistically significant decline in PHQ-2 score (1.03 to 0.19; *P*=.01), while type 1, type 2, and participants not using insulin saw a nonstatistically significant improvement in scores.

## Discussion

### Principal Findings

The results of this study in an outpatient diabetes clinic provide evidence that access to a cellular-enabled BG meter connected to CDEs with real-time personalized recommendations can improve HbA_1c_. This improvement was significant since our study showed that with lower HbA_1c_, participants also had increased BG values within target range, with a decrease in hypoglycemic events at 12 months. Furthermore, study participants had improved depressive symptom scores as measured by PHQ-2 surveys. Overall, a connected BG meter with personalized feedback and access to CDEs improved diabetes care at 12 months.

The ADA and WHO recognize digital health and technology advances can support and enhance the delivery of health services [6,19]. Specifically, the ADA’s 2019 Standards of Medical Care includes recommendations for diabetes technology recognizing digital self-management solutions for improvement in HbA_1c_, especially when paired with a health care team, individualized feedback, patient generated historical health data, and education [19]. Additionally, the ADA recommends patients receive ongoing education and evaluation of glucose data to adjust therapy and self-care in relation to individual needs [19,22]. With the growing global epidemic of diabetes linked to a lack of knowledge around BG monitoring, self-management, education, and episodic communication with health care providers, testing a cellular-enabled BG meter with BG checking reminders, personalized digital coaching, access to CDEs, and historical BG reporting was critical to understand the benefit of including digital diabetes solutions related directly to issues increasing diabetes prevalence, in addition to a diabetes clinic’s standard care [1-5].

Using a cellular-connected BG meter provides health care professionals with instant access to patient-generated BG readings, allowing for faster change in care plan, education, and outcomes. In addition, a patient’s care team can provide a more personalized and proactive plan, with tailored education through the system’s generated insights of historical BG data and two-way communication with CDEs available through the Livongo meter [23]. Viewing a patient’s BG values within low, normal, or high ranges over a week or month allows for a timelier response in condition management versus waiting 3 months for a change in HbA_1c_. In addition, two individuals with the same HbA_1c_ could have very different time in BG ranges, which would impact desired treatment plan. Without the ability to view historical BG readings in a timely manner, an important aspect of an individual’s personalized care plan could be overlooked as it would not be reflected in HbA_1c_. As such, our study supports previous findings that access to a cellular-connected glucometer and CDE coaching decreases hypoglycemic episodes and leads to a decrease in HbA_1c_ up to 1% in 3 months [11,13,15]. Also, having access to a program like Livongo for Diabetes can provide continued BG monitoring, education, and coaching for individuals with diabetes who choose to not follow up with their health care team as recommended.

Combining digital health tools with human coaching for individuals with diabetes has also improved depressive symptoms, as measured by PHQ scores [17,18,24]. By incorporating technology and access to real-time support from CDEs into diabetes care, the challenges of self-management that can increase depressive symptoms, such as support, emotional burden, and access to education and management, are addressed in a more timely manner focused on the patient’s personalized needs [17,25]. The addition of coaching offers reinforcement of education, accountability, and creation of problem-solving skills to overcome behavioral and cognitive barriers for successful self-management [25].

The Livongo for Diabetes program has also been shown to provide cost savings to its users [26,27]. In 2019, Livongo users had a 21.9% decrease in spending compared to nonusers, translating to $88 per month. Specifically, a 10.7% reduction was observed in diabetes-related medical spending, and a 24.6% reduction in spending for office-based services [27]. While historically offering ongoing human coaching can be costly, the Livongo for Diabetes program has provided a return on investment for its users while improving clinical outcomes.

### Limitations and Future Research

The limitations of this study include the lack of a control group and the small sample size for subgroup comparisons. Authors assume the dramatic decline in sample size is related to a physician leaving the clinic, resulting in a lack of follow up from that physician’s patients since patients come to the clinic to see specific providers. In addition, participants for this study were recruited at a Diabetes Center of Excellence, which provides access to the highest level of diabetes care. The BG checking frequency was unexpectedly low in the type 1 diabetes population. This finding may be a result of continuous glucose monitor use in this population, which requires two BG checks for calibration. Neither this information nor the use of non-Livongo BG meters were captured as part of the study.

Finally, changes in medication use, weight, knowledge of diabetes self-management, coaching interactions, and other factors that might influence BG control were not captured as part of this study. Further investigations will be required to see if findings would be applicable to the general population and to better understand the drivers of improved glucose control.

Despite the small sample size, this study provides a glimpse of how adding a new product into the market, or with standard care, can improve patient outcomes even in centers of excellence. This is an important contribution to the literature and for larger population studies in the future.

### Conclusions

Participants provided with a cellular-enabled BG meter with real-time feedback and access to CDE coaching in a diabetes center of excellence experienced a reduction in HbA_1c_, fewer hypoglycemic episodes, and a significant reduction in PHQ-2 scores. These results support evidence that the addition of diabetes digital health solutions can improve diabetes care. Further studies should be conducted to assess a larger population with the addition of coaching interactions, medication use, education, and self-management behaviors.

## Appendix

Multimedia Appendix 1 Livongo Diabetes Glucose Meter.

Multimedia Appendix 2 Livongo diabetes smartphone app.

Multimedia Appendix 3 BG checks in range by diabetes type from 0-3 months to 9-12 months.

## Abbreviations

AADE: American Association of Diabetes Educator
ADA: Americans with Disabilities Act
BG: blood glucose
CDE: certified diabetes educator
HbA_1c_: hemoglobin A_1c_
HSR: health summary report
PHQ: Patient Health Questionnaire
USH DHHL: University of South Florida Diabetes Home for Healthy Living
WHO: World Health Organization
