# Figure Correction: The Effect of a Cellular-Enabled Glucose Meter on Glucose Control for Patients With Diabetes: Prospective Pre-Post Study

**DOI:** 10.2196/21993

**Published:** 2020-07-28

**Authors:** Jennifer B Bollyky, Stephanie T Melton, Tong Xu, Stefanie L Painter, Brian Knox

**Affiliations:** 1 Stanford University School of Medicine Stanford, CA United States; 2 University of South Florida Florida, CA United States; 3 Livongo Health Mountain View, CA United States

In “The Effect of a Cellular-Enabled Glucose Meter on Glucose Control for Patients With Diabetes: Prospective Pre-Post Study” (JMIR Diabetes 2019;4(4):e14799), the authors noted an error in the caption of Figure 6.

The caption formerly said:

Change in hemoglobin A_1c_ (HbA_1c_) from baseline by timepoint for type 2 diabetes with insulin use.

This has been revised to:

Change in hemoglobin A_1c_ (HBA_1c_) from baseline by timepoint for all participants with insulin use.

The correction will appear in the online version of the paper on the JMIR Publications website on July 28, together with the publication of this correction notice. Because this was made after submission to PubMed, PubMed Central, and other full-text repositories, the corrected article has also been resubmitted to those repositories.

