# Supplementary figures and images for: The Effect of a Cellular-Enabled Glucose Meter on Glucose Control for Patients With Diabetes: Prospective Pre-Post Study
Source: JMIR Diabetes. 2019 Oct 7;4(4):e14799. doi: 10.2196/14799 (PMC6803884; doi:10.2196/14799)

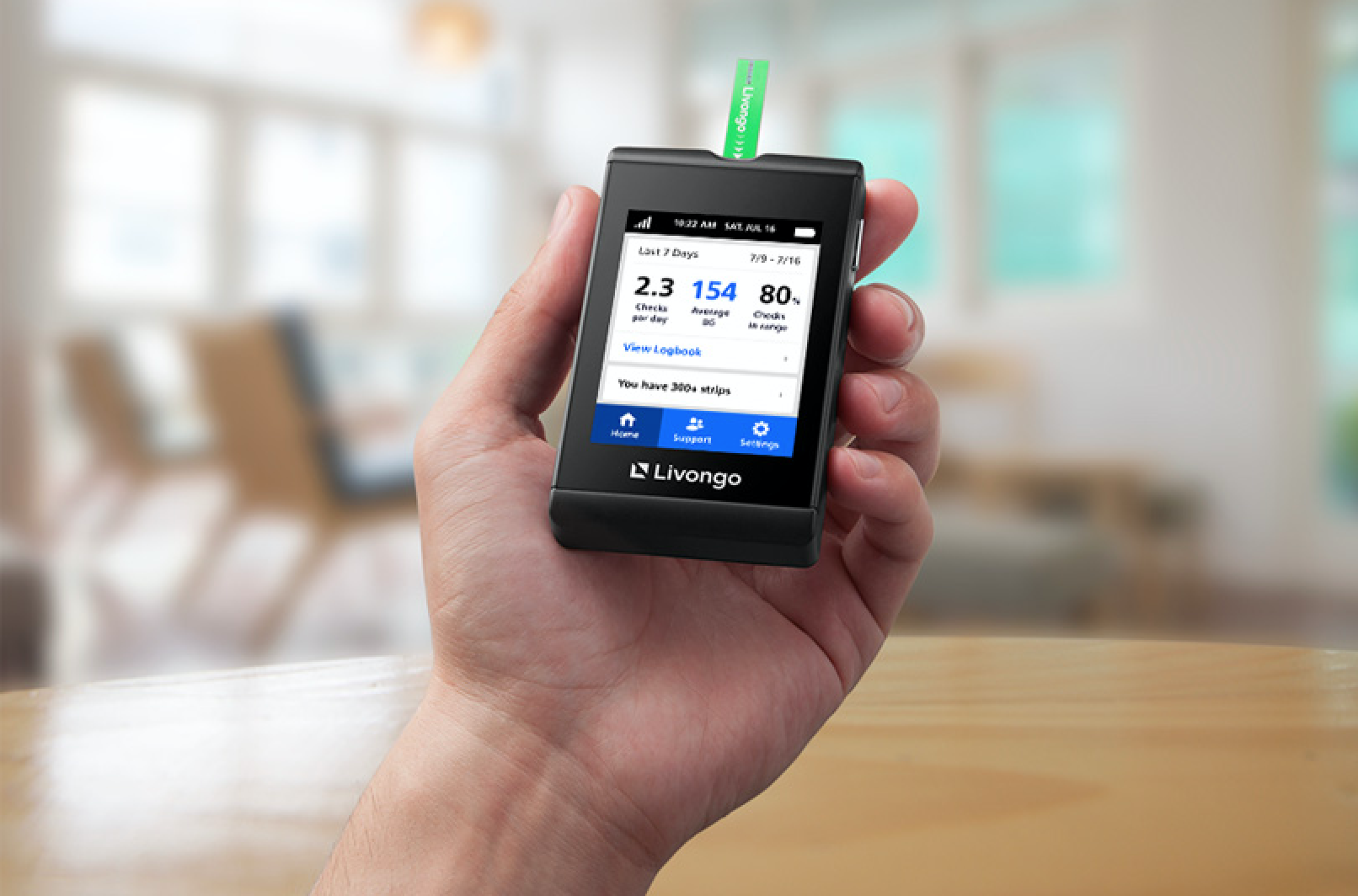

Supplement: Multimedia Appendix 1 [file diabetes_v4i4e14799_app1.png]

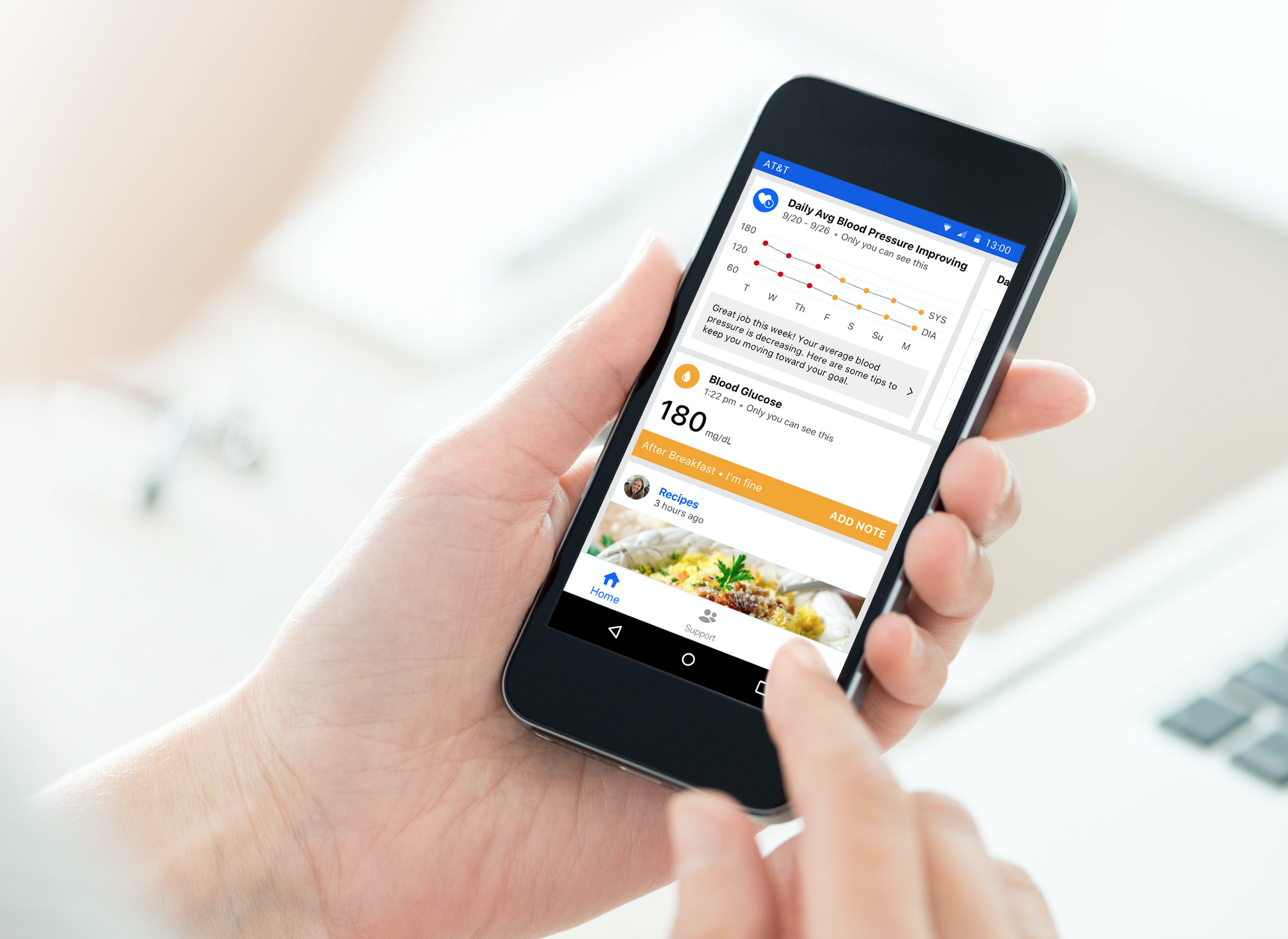

Supplement: Multimedia Appendix 2 [file diabetes_v4i4e14799_app2.png]
