# Supplementary material for: The Effect of a Cellular-Enabled Glucose Meter on Glucose Control for Patients With Diabetes: Prospective Pre-Post Study
Source: JMIR Diabetes. 2019 Oct 7;4(4):e14799. doi: 10.2196/14799 (PMC6803884; doi:10.2196/14799)
Supplement: Multimedia Appendix 3 [file diabetes_v4i4e14799_app3.pdf]

S1 APPENDIX

Diabetes Type	Type 1				Type 2 with Insulin				Type 2 without Insulin				All			
Time in Range	0-3 M		9 - 12 M		0-3 M		9 - 12 M		0-3 M		9 - 12 M		0-3 M		9 - 12 M	
	N	SD	N	SD	N	SD	N	SD	N	SD	N	SD	N	SD	N	SD
Number of Participants	37		18		20		16		26		18		83		52	
BG checks per person, mean	139	121	186	184	146	91	120	116	129	127	85	71	137	115	131	137
BG Value, mean	157	43	173	48	173	56	159	52	131	26	138	38	153	45	156	48
% BG checks <54 mg/dL	3.8%	4.6%	3.7%	6.5%	0.1%	0.2%	0.2%	0.5%	0.0%	0.0%	0.0%	0.0%	1.7%	3.6%	1.3%	4.1%
% BG checks 54-70 mg/dL	6.2%	6.3%	5.5%	6.7%	0.4%	0.6%	1.1%	2.4%	1.2%	2.2%	1.5%	3.1%	3.2%	5.1%	2.8%	4.9%
% BG checks 70-180 mg/dL	59.5%	23.1%	50.2%	22.5%	65.9%	30.8%	72.8%	29.5%	89.6%	13.7%	86.1%	22.2%	70.5%	26.2%	69.6%	28.6%
% BG checks 181-250 mg/dL	18.4%	11.1%	22.8%	11.6%	18.9%	15.7%	16.1%	15.3%	8.6%	12.4%	7.9%	11.4%	15.4%	13.4%	15.5%	14.0%
% BG checks 251-400 mg/dL	10.4%	11.8%	16.5%	16.7%	12.6%	18.3%	8.7%	16.0%	0.6%	1.7%	4.5%	15.8%	7.8%	12.9%	9.9%	16.6%
% BG checks > 400 mg/dL	1.8%	5.4%	1.5%	3.5%	2.1%	4.9%	1.2%	3.3%	0.1%	0.3%	0.0%	0.0%	1.3%	4.3%	0.9%	2.8%
